# A Decision Framework for Selecting Critically Important Nutrients from Aquatic Foods

**DOI:** 10.1007/s40572-023-00397-5

**Published:** 2023-05-25

**Authors:** Jessica Zamborain-Mason, Daniel Viana, Khristopher Nicholas, Erin D. Jackson, J. Zachary Koehn, Simone Passarelli, Seo-Hyun Yoo, Angela W. Zhang, Hannah C. Davin, Christopher P. Duggan, Josef Schmidhuber, Christopher D. Golden

**Affiliations:** 1 Department of Nutrition, Harvard T.H. Chan School of Public Health, Boston, MA 02115 USA; 2 Division of Agriculture, Food, and Environment, Friedman School of Nutrition Science and Policy, Tufts University, Boston, MA 02111 USA; 3 Stanford Center for Ocean Solutions, Stanford University, Stanford, CA 94305 USA; 4 Office of Global Food Security, U.S. Department of State, Washington, D.C 20502 USA; 5 Emerson Fellowship, Congressional Hunger Center, Washington, D.C 20502 USA; 6 Department of Environmental Health Sciences, Mailman School of Public Health, Columbia University, New York, NY 10032 USA; 7 Center for Nutrition, Division of Gastroenterology, Hepatology and Nutrition, Boston Children’s Hospital, Boston, MA 02115 USA; 8 Markets and Trade Division, FAO, Rome, Italy

**Keywords:** Macronutrients, Micronutrients, Public health, Planetary health, Blue foods, Fisheries, Aquaculture, Food and nutrition security

## Abstract

**Purpose of Review:**

Aquatic foods are increasingly being recognized as a diverse, bioavailable source of nutrients, highlighting the importance of fisheries and aquaculture for human nutrition. However, studies focusing on the nutrient supply of aquatic foods often differ in the nutrients they examine, potentially biasing their contribution to nutrition security and leading to ineffective policies or management decisions.

**Recent Findings:**

We create a decision framework to effectively select nutrients in aquatic food research based on three key domains: human physiological importance, nutritional needs of the target population (demand), and nutrient availability in aquatic foods compared to other accessible dietary sources (supply). We highlight 41 nutrients that are physiologically important, exemplify the importance of aquatic foods relative to other food groups in the food system in terms of concentration per 100 g and apparent consumption, and provide future research pathways that we consider of high importance for aquatic food nutrition.

**Summary:**

Overall, our study provides a framework to select focal nutrients in aquatic food research and ensures a methodical approach to quantifying the importance of aquatic foods for nutrition security and public health.

**Supplementary Information:**

The online version contains supplementary material available at 10.1007/s40572-023-00397-5.

## Introduction

Aquatic foods have historically been considered a critical source of protein intake. However, increasing evidence suggests that aquatic foods are also a vital source of essential fatty acids and key micronutrients [1–3]. As a consequence, studies have increasingly focused on the contribution of animal-sourced aquatic foods to nutrition and public health [4•, 5], the potential for fisheries and/or aquaculture to contribute to nutrition security [6, 7•, 8–10], and pathways to manage and conserve aquatic foods in nutrition-sensitive ways to prevent prevalent nutrient deficiencies [11•, 12–15].

Recent compilations of aquatic food nutrient composition data on a diverse range of aquatic species [4•, 16] have expanded the scope of research questions that can be addressed on how aquatic foods contribute to nutrition security. For example, models have been developed that predict the nutrient composition in finfish using either phylogenetic traits [6] or species-specific environmental and life history characteristics [7•]. The latter model outputs have been integrated into FishBase [17], making nutrient composition estimates available for all fish species (many with missing observed data) in a global database commonly used by ichthyologists, ecologists, and fisheries scientists.

While the assembly of aquatic food composition databases and the development of nutrient predictions for missing species have been key steps towards understanding the potential of aquatic foods for nutrition security (i.e., consistent access, availability, and affordability of foods and beverages that promote well-being, prevent disease, and, if needed, treat disease [18]), there remains uncertainty in selecting nutrients to prioritize in modeling and optimization exercises. Food composition tables are region-specific repositories of nutrition content for food that include many nutrients. But how does one select which nutrients to use in aquatic food and nutrition studies? For example, the concentration of vitamin B_12_ (i.e., cobalamin) is considered high in aquatic foods [4•] but the nutrient is missing from models that predict fish nutrient composition from environmental and life-history fish traits [7•], and from studies aiming to develop nutrition-sensitive reference points for fisheries or emphasizing the role of fisheries management for nutrition [11•, 12]. Additionally, selenium, which is often available in seafood sources [19], is missing from studies comparing the nutrient contribution of aquatic foods to other food groups (e.g., [4•]). Furthermore, some nutrients (e.g., folate) that are both important for human health [20] and rich in aquatic food sources relative to other animal-source products [21] are missing entirely from such studies (e.g., [4•, 7•]). The selection of nutrients could influence combined nutrient metric conclusions (e.g., sustainable exploitation rates to maximize overall nutrient output or pooled micronutrient density scores; e.g., [11•, 22]) and potentially misrepresent the role of aquatic food resources for nutrition security.

**Fig. 1:**
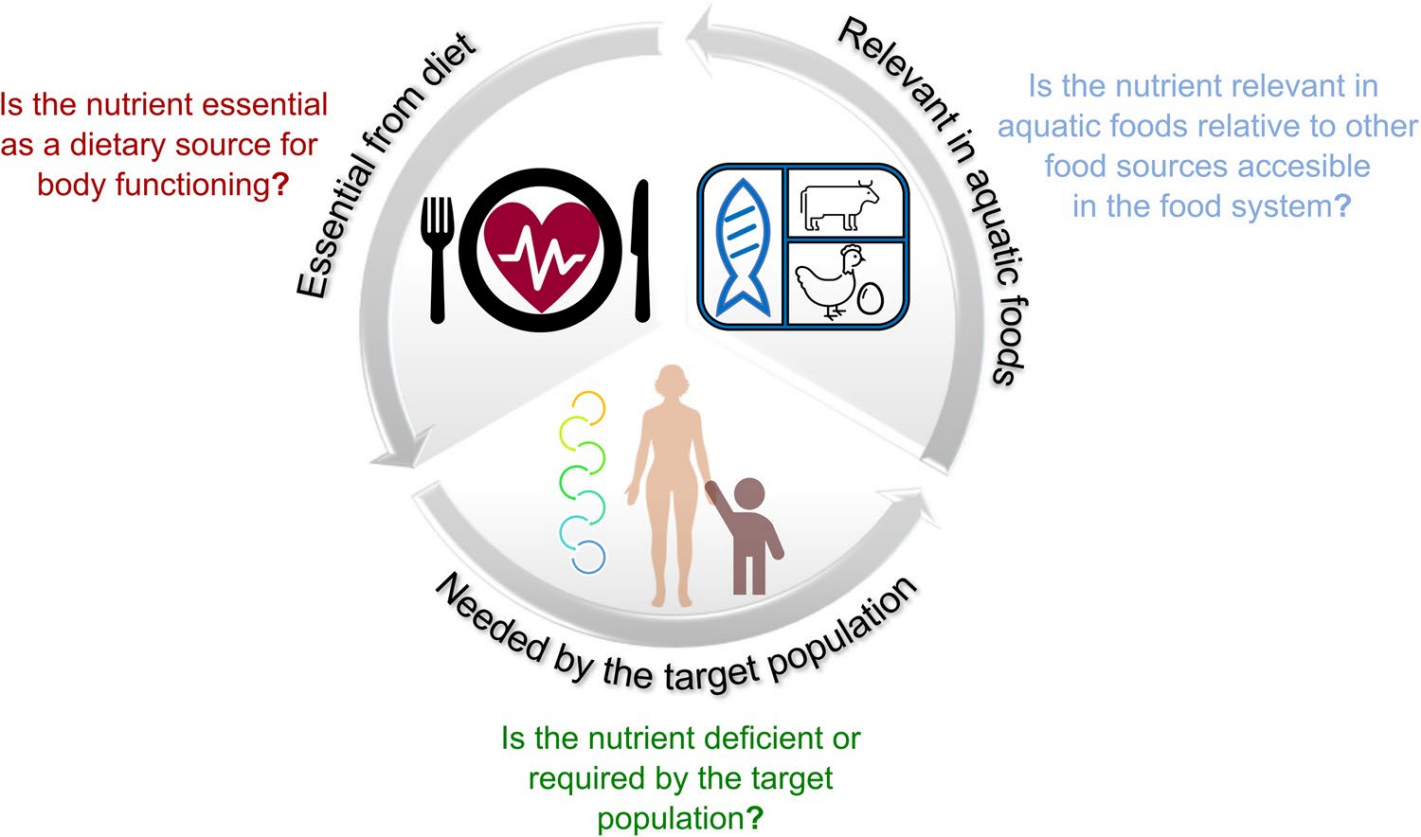
Diagram of a decision framework to select nutrients in aquatic food research

In this review, we propose a framework to select nutrients based on (i) human physiological importance, (ii) nutritional requirements (demand) of the target population, and (iii) nutrient concentration and total availability of aquatic foods in comparison to other dietary sources accessible in the food system (Fig. 1). First, we narrow our nutrient pool to focus only on nutrients that are obtained from dietary sources and are essential for human nutrition. Second, we consider the context of study populations to determine which nutrients must be supplied to avoid inadequate nutrient intake and deficiencies. Third, we compare different food groups in terms of nutrient concentration per 100 g of food and estimated availability (i.e., apparent consumption) to emphasize the importance of accounting for other accessible food sources in the food system when selecting nutrients in aquatic food research. Finally, using case studies, we provide examples of how one may follow this framework to select nutrients more effectively. Our study proposes a methodological approach to nutrient selection for aquatic food research and highlights future data and research needs to continue unlocking the role of aquatic foods for nutrition security.

## Identifying Nutrients Important for Public Health

**Fig. 2:**
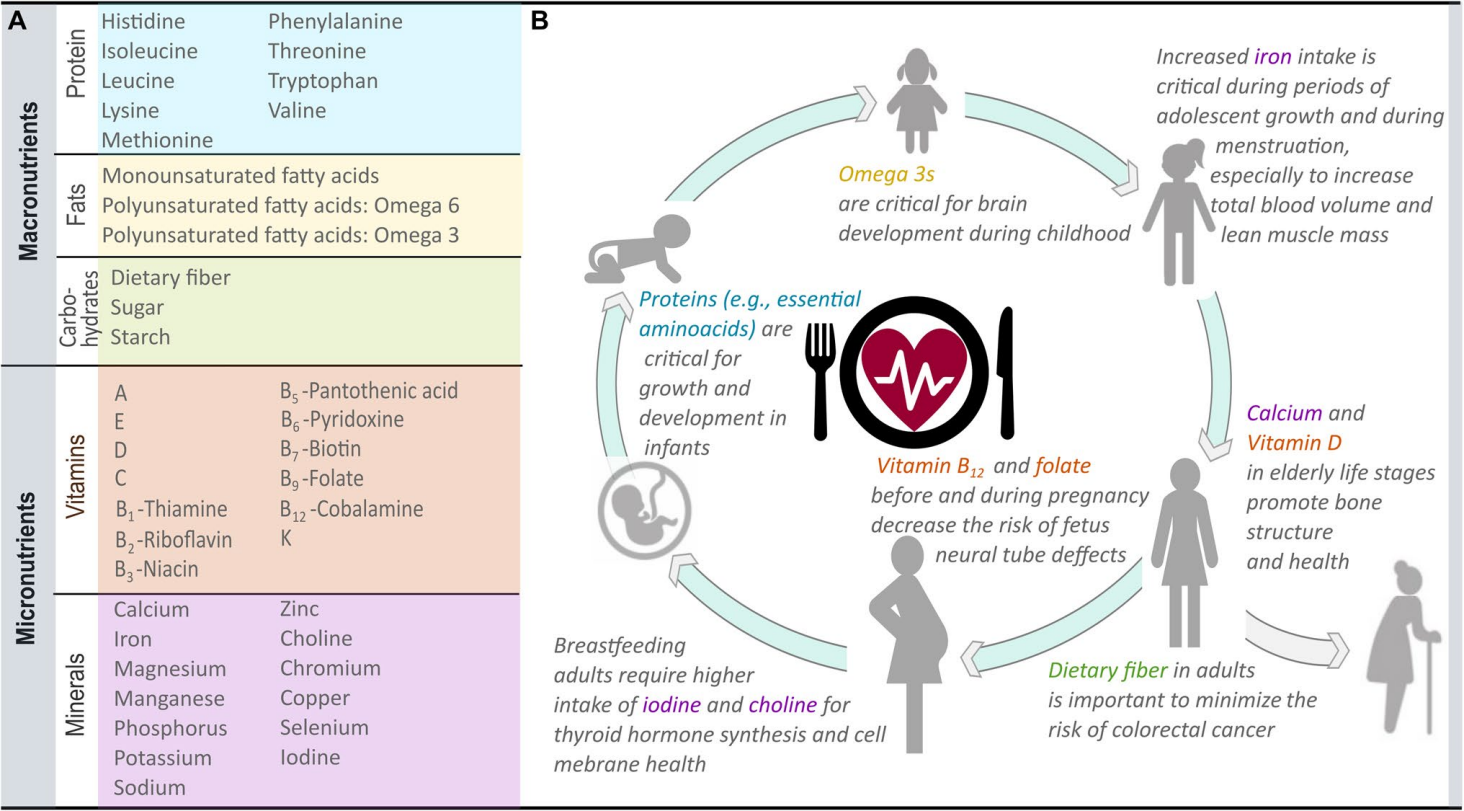
Diagram identifying key nutrients for physiological functioning from dietary sources. **A** All nutrients identified from dietary sources that are required for proper human body functioning. **B** Examples on nutrient benefits for different human life stages

First, we reviewed the nutrients essential for human physiological functioning that are obtained from dietary sources. We found that, based on current evidence, there are a total of 41 nutrients sourced from the diet that are essential for physiological functioning (Fig. 2; Table S1) [23••].

Nutrients can be divided into macronutrients and micronutrients. Macronutrients—protein, carbohydrates, and fats—are the nutritious components of food that must be consumed in large quantities to maintain the body’s energy needs, structure, and metabolic functioning [23••]. Macronutrients are divided into subgroups. For example, protein is composed of amino acids. Of the 20 existing amino acids, nine (histidine, isoleucine, leucine, lysine, methionine, phenylalanine, threonine, tryptophan, and valine) are essential, as they are not synthesized by the body and must be consumed in food [24]. Carbohydrates are divided into fiber, simple sugar, and starch, all of which are important energy sources to the human body. Fats are made of fatty acids, which can be in the form of saturated fatty acids, monounsaturated fatty acids, and polyunsaturated fatty acids. Polyunsaturated fats include omega-6 and omega-3 fatty acids. Omega 3 fatty acids include alpha-linoleic acid (ALA), docosahexaenoic acid (DHA), and eicosapentaenoic acid (EPA) which are crucial for brain development and cardiovascular health [25, 26] and are found in high concentrations in aquatic food sources [27].

Micronutrients—vitamins and minerals—must be consumed in trace amounts for proper physiological function, growth, and development [28]. Vitamins are organic compounds synthesized by plants and animals while minerals are inorganic compounds absorbed from soil and water [23••]. There are at least thirteen vitamins and thirteen minerals required from the diet that enable proper physiological functioning and minimize the risk of non-communicable diseases [24, 29]. For an overview of key nutrients, their biological importance, main dietary sources, and examples of deficiency consequences see Table S1.

## Accounting for the Target Population and Their Nutritional Needs

After narrowing the nutrient pool to those sourced from the diet and essential for human physiological needs (e.g., Fig. 2), we suggest considering the target population’s need (i.e., demand) for particular nutrients (e.g., [30]) given their prevalence of deficiency (e.g., geographical context), and/or biological need (e.g., based on demography). Ideally, one would have a representative measure of the target population’s health and prevalence of deficiency, such as temporal trends in biomarkers [31, 32]. However, in the absence of direct deficiency measurements, other available proxies of nutritional needs, like inadequate intake [33], may be combined with local context (e.g., demography and geography) to inform aquatic food research.

**Fig. 3:**
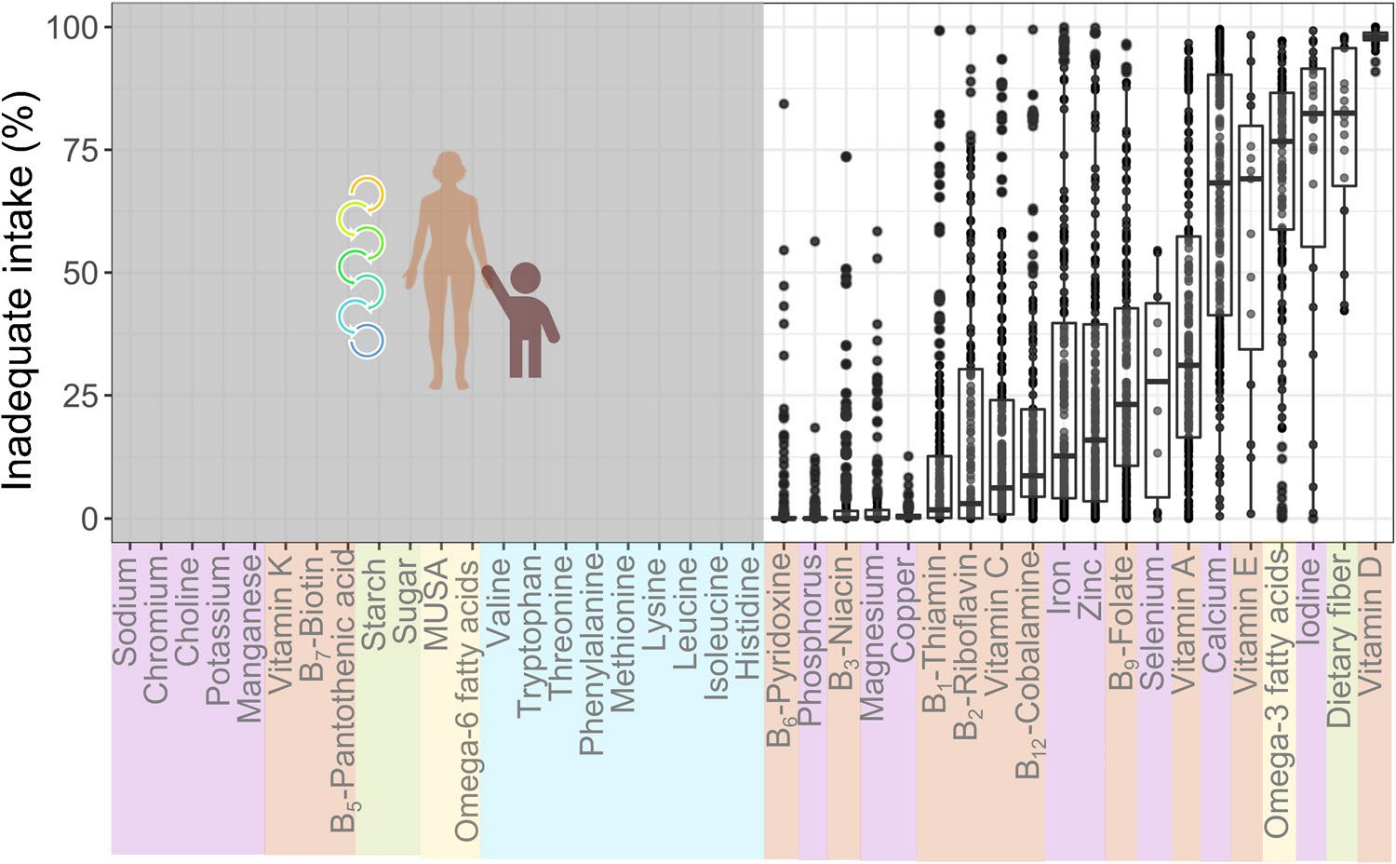
Estimated global inadequate dietary intake. Each point is a country’s mean inadequate intake across age/sex groups and considered global studies [4•, 33, 37, 38]. Nutrients without information are those that do not have inadequate intake estimates within the considered global studies (here shaded in gray)

Inadequate intake (e.g., Fig. 3), which is based on the supply and apparent consumption of available foods, is commonly used to measure the nutrient intake of a population and risk of nutrient deficiencies [34, 35]. Globally, there is widespread inadequate intake of vitamins and minerals, often geographically co-occurring [36]. For example, by combining available data of inadequate intake studies at global scales [4•, 33, 37, 38], we found that vitamin D is estimated to be the nutrient with the highest inadequate dietary intake globally, followed by fiber, iodine, omega three fatty acids, vitamin E, calcium, vitamin A, selenium, thiamine, zinc, iron, and vitamin B_12_ (Fig. 3). Thus, these nutrients with a high risk of deficiency may be prioritized for aquatic food research with a global focus if direct deficiency estimates are not available.

Population nutritional requirements vary based on demography, with some nutrients becoming particularly important in specific human life stages or demographics (Fig. 2). Adequate prenatal folate intake, for example, is critical to lower the risk of fetal neural tube malformations [20], whereas iron intake is critical for adolescent women in menstruation phases to increase total blood volumes [39]. Similarly, breastfeeding adults require higher intakes of iodine and choline to promote thyroid synthesis and membrane health [40], whereas adults in their elderly life stages require higher intakes of vitamin D and calcium to prevent bone diseases (Fig. 2). Overall, if one were to select nutrients for a more localized study without accounting for the local population demography, accurate population requirements based only on national-level inadequate intake estimates may be misrepresented, and resulting aquatic food programs and policies may not be as effective in achieving their goal.

Population requirements also vary based on geographical context. Some locations may have higher or lower risk of nutrient deficiencies than those highlighted by inadequate intake due to other related causes [41]. Vitamin D deficiencies, for example, are estimated to affect 1 billion people worldwide [42]. However, vitamin D, besides being obtained from dietary sources, is also synthesized by the human body from sunlight. Thus, studies focusing on aquatic foods for nutrition security in regions that receive enough vitamin D from sunlight year-round (e.g., some tropical coral reef regions), may not need to optimize aquatic foods for such a nutrient, even if dietary intake data may suggest vitamin D inadequacy. Those living in temperate and polar regions, on the other hand, are likely receiving insufficient sunlight to satisfy their vitamin D requirements [43]; thus, inadequate intake may indeed be addressed through consumption of aquatic foods in some seasons. Similarly, iron and zinc deficiencies are believed to increase in areas of high infectious disease burden due to decreased absorption, even if nutrient intake levels are adequate [44]. The prevalence of anemia, for example, can also be associated with a deficiency in iron, vitamin A, vitamin B_12_, and/or folate [45, 46]. Estimating the likelihood of nutritional vulnerability of a study population that does not have direct deficiency estimates may therefore require combining dietary intake data and/or other common proxies for nutrient deficiencies.

## Positioning Aquatic Foods Relative to Other Accessible Nutrient Sources

To understand the potential contribution of aquatic foods to nutrient supply and to determine which nutrients to select for aquatic food nutrition, we must also account for both the nutrient concentration and total availability of other dietary sources that are culturally acceptable and affordable to the population, as well as available supplementation and fortification in a given context. In other words, for a given nutrient required by the population, are aquatic foods appropriate nutrient sources or are other accessible dietary sources in the food system more appropriate? To exemplify this, we ranked the concentrations and apparent consumption of each essential nutrient among different food sources using the Aquatic Food Composition Database [4•, 47], the United States Department of Agriculture (USDA) food composition tables [48], and nutrient apparent consumption estimates [49••] (NB: if available, food composition tables relevant to the study location should be considered [50]). This process allowed us to emphasize that a key step when prioritizing nutrients in aquatic food research is to consider other food and/or nutrient sources in the food system and their potential contribution to nutrient supply and intake in terms of both nutrient concentration and food quantity (including how prevalent their consumption is).

**Fig. 4:**
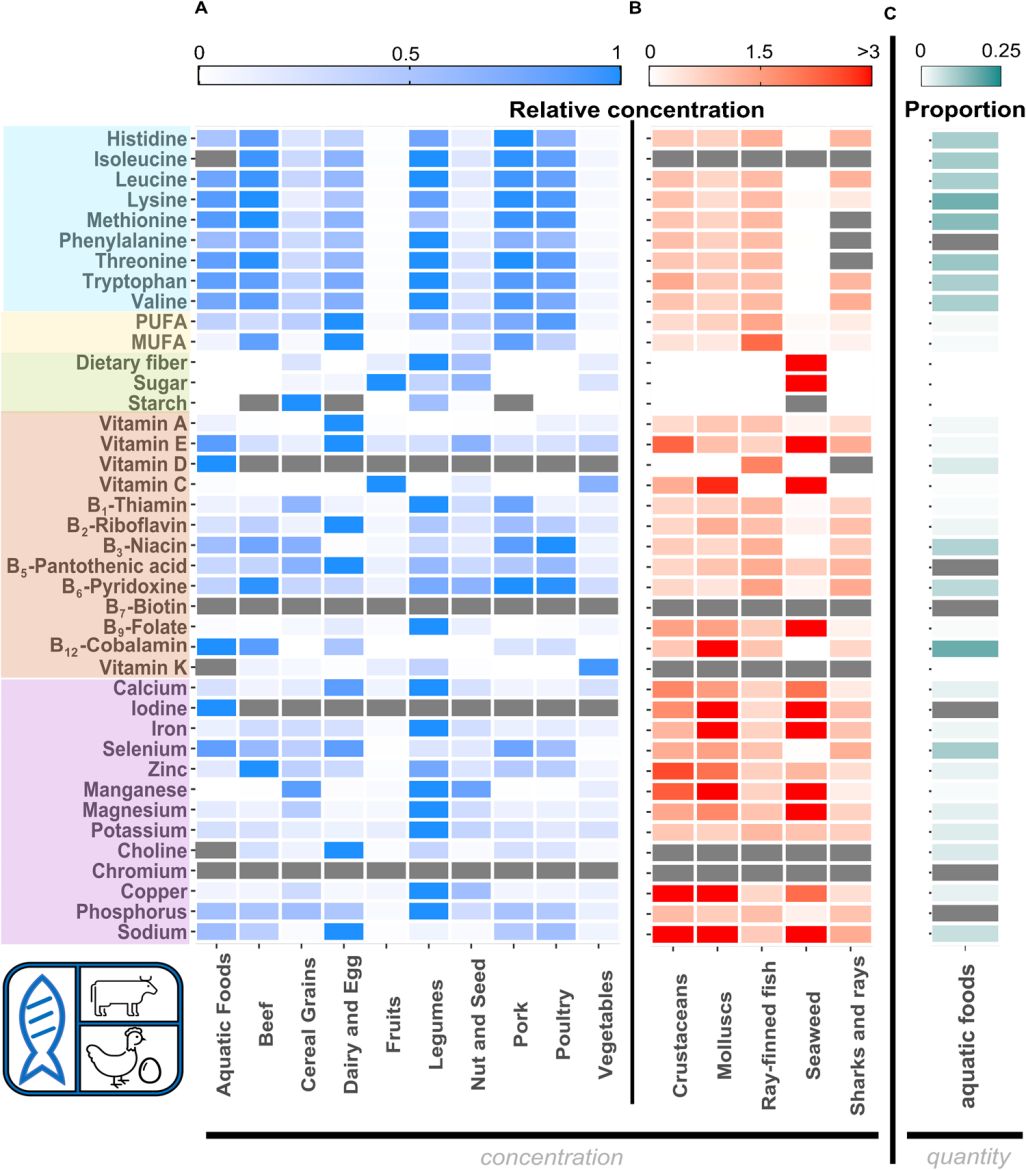
Ranking of aquatic foods relative to other food groups. **A** Aquatic foods relative to other food groups in terms of concentration per 100 g of raw product. Raster map is based on median values and scaled according to the food group with the highest median concentration per 100 g. See supplementary information Figure S1 to see within-group variability. **B** Within aquatic food group variability. The raster map is based on median concentration per 100 g raw muscle tissue values and scaled according to the median concentration of all aquatic foods jointly. **C** Proportion of median per-capita nutrient apparent consumption obtained from aquatic foods relative to the total diet. In **A**–**C**, gray values indicate nutrients without data in the databases we examined. Note that in this section omega 3 and omega 6 (e.g., Fig. 2) are combined into polyunsaturated fatty acids (i.e., PUFA) due to data availability. MUFA refers to monounsaturated fatty acids

In terms of nutrient concentrations, we show that, based on median raw muscle tissue values, aquatic foods rank higher than all other foods for iodine, vitamins D, and B_12_ (Fig. 4). This suggests that aquatic foods, when available and affordable, are ideal candidates to tackle such nutrient deficiencies. In contrast, aquatic foods were not ranked highly as a source of vitamin A. However, if other vitamin A-rich food sources (e.g., dairy and eggs in Fig. 4) are not accessible to the study population (e.g., not available in sufficient quantities or not affordable), aquatic foods may become a critical source. This highlights the need to consider aquatic foods relative to other accessible foods.

Intra-food group variability (e.g., which specific aquatic foods) also matters when we select nutrients. For example, within aquatic foods, bivalves had the highest concentrations per 100 g of raw muscle tissue in 10 out of the 40 nutrients examined with available data, whereas crustaceans were relatively higher than all other aquatic foods in copper, phosphorus, sodium, tryptophan, and zinc (Fig. 4). This within-group variability also applies to non-aquatic foods (Fig. S1). For example, within vegetables, leafy greens—such as kale and spinach—have a much higher concentration of vitamin K compared to carrots or cruciferous vegetables like cauliflower [51]. Thus, it is critical to consider the availability of alternative specific food sources, how specific food groups are combined (e.g., dietary patterns), and how aquatic foods can best contribute towards addressing specific population nutritional needs.

To evaluate total nutrient supply and the relative importance of aquatic foods, one must account for the product of concentration and quantity of each available and affordable food type (i.e., not only what food types are accessible, but in what quantities and how prevalent their consumption is in the diet). For instance, although rice does not have a high concentration of zinc, the sheer volume of consumption in countries like Bangladesh and Madagascar causes rice to be the primary source of zinc to the population [52]. Based on median concentration values, aquatic foods did not rank the highest in 90% of the nutrients we examined. However, when we account for apparent consumption of such foods [49••], which includes quantity, aquatic foods were estimated to contribute > 10% of the per-capita apparent consumption for vitamin B_12_, selenium, and all essential amino acids with available information (based on median values from 193 countries; Fig. 4c). Therefore, when selecting nutrients for aquatic food nutrition research, which foods are available and affordable in the food environment, in what quantities and how prevalent their consumption is in the diet, is an important step to understand (i) which nutrients aquatic foods could contribute the most to, and (ii) what nutrients are obtained from other foods, supplementation or fortification so aquatic foods can complement them.

## Examples of Nutrient Selection in Aquatic Food Research

**Fig. 5:**
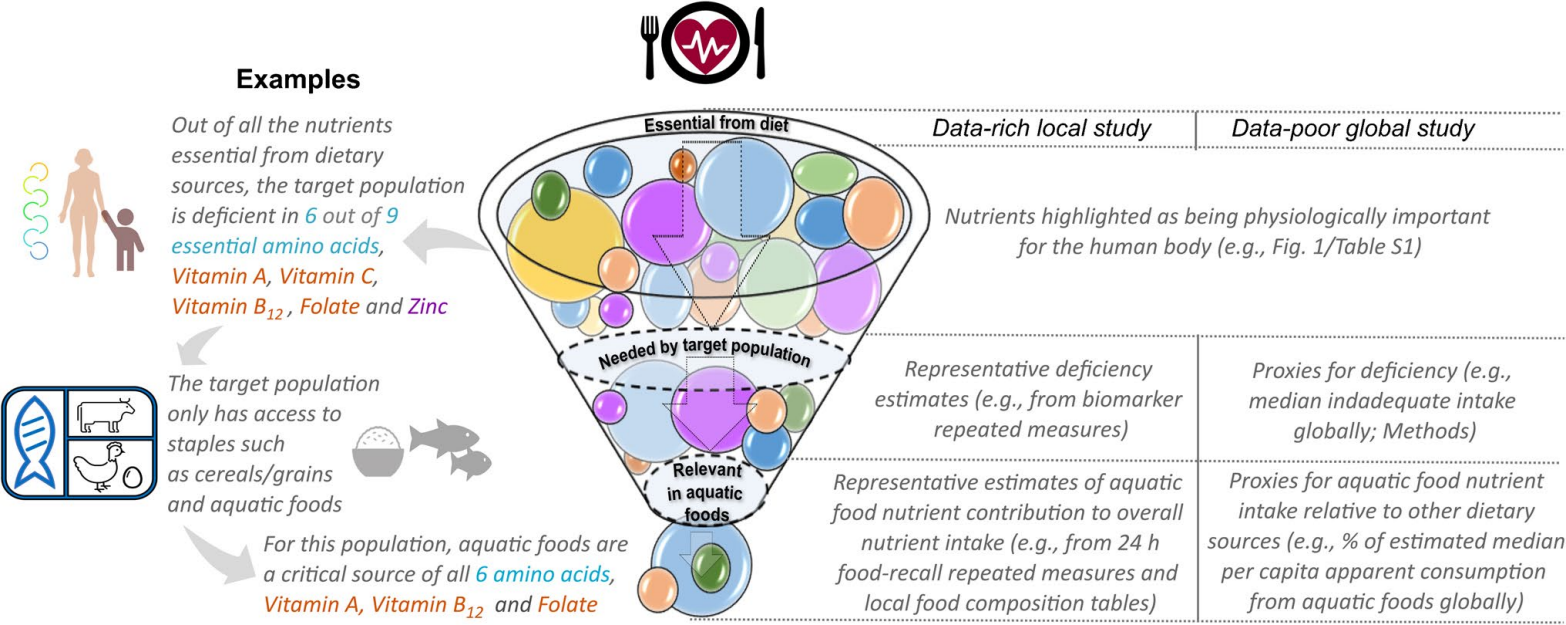
Decision framework for nutrient selection in aquatic foods research. We provide an example of how one may prioritize nutrients in aquatic food research under a given context (left side of filter diagram) and potential pathways to operationalize nutrient selection depending on data availability (right side of filter diagram)

Here, we provide several examples to select nutrients in aquatic food research under different data-availability scenarios (Fig. 5). Imagine you are conducting aquatic food nutrition research in a tropical coastal community to understand how local fisheries can be managed to optimize nutrient supplies and improve population health (e.g., [11•, 53]). The first question one might ask is: which dietary source nutrients are essential for the population? The answer to this question will provide a wide list of nutrients that are physiologically required (e.g., Fig. 2). The second question is: which nutrients are at risk of deficiency in the population given their characteristics? Given the location of the study population, one may measure their current deficiency status (e.g., with biomarker repeated measures) and/or, if such data is not available, evaluate the population’s demography and/or use proxies (e.g., nutrient inadequate intake, anemia or stunting) that give a measure of nutrient deficiency risk. Such a process may reveal that the population is deficient (or at risk of deficiency) in protein, missing six out of nine essential amino acids in sufficient quantities, vitamins A, C, and B_12_, folate, and zinc (e.g., Fig. 5). One may initially aim to consider these nutrients. However, there are likely nutrient trade-offs, with some management measures optimizing one nutrient but not others. For example, harvesting fish stocks at different rates maximizes different nutrient yields depending on the species mix [11•]. Consequently, the most salient research question may actually be as follows: to which nutrient intakes can aquatic foods contribute most given the existing food environment? At such a stage other accessible food sources come into play: which foods does the population have access to and in what quantities are they consumed? For our coastal community example, cereals/grains and aquatic foods are the primary food sources, with cereals/grains consumed in larger quantities. Food composition tables and intake estimates (e.g., from repeated food recalls or apparent consumption) reveal that available cereals and grains are richer in zinc and vitamin C and contribute the highest percentage to those nutrient intakes in comparison to accessible seafood resources, whereas aquatic foods contribute most to all other nutrients the population is deficient in. Thus, one may choose to prioritize research on the six essential amino acids, vitamin A, vitamin B_12_, and folate, which are (i) essential for the population from dietary sources, (ii) deficient (or at risk of deficiency) in the target population, and (iii) most relevant in aquatic foods in comparison to other accessible food sources (i.e., cereals and grains).

Of course, food access and dietary needs of a population may change over time, and such dynamics require timely re-evaluation of nutrient selection. For example, one may also ask: is access to current food resources and quantities stable and/or sustainable? In our case study, imagine freshwater resources used to cultivate available cereals and grains become scarce due to ongoing reduction in precipitation with climate change [54]. Cereals and grains may still be accessible to the population but in much lower quantities that are insufficient to satisfy the population’s zinc requirement [55]. In such a case, available aquatic resources may become a critical source of zinc for the population in the medium to long term.

## Frontiers to Improve Nutrition-Based Aquatic Food Research

Our study proposes a methodological and evidence-based decision framework to select nutrients in aquatic food research. The established criteria within the framework vary by space and time and we suggest considering nutrient targets at the start of the sampling design of any nutritional research on aquatic foods. Furthermore, below, we outline six future research avenues that we consider of high importance to further inform nutrient selection and understand the contribution of aquatic foods to nutrition security.

### Improving Nutrient Composition Data

Often, nutrient selection in aquatic food research is driven by data availability (e.g., [7•]). Many food composition tables are limited in the nutrients reported relative to those highlighted in Fig. 2 (e.g., [56]) or are biased towards foods and countries that have better monitoring and/or reporting (e.g., [48]). For aquatic foods specifically, several datasets exist, however each with its own benefits and caveats [57], and with limited spatio-temporal resolution. Strategically allocating research efforts to collect baseline food composition data that is spatially and temporally representative, especially for nutrients that have been under sampled (e.g., isoleucine or vitamin K in aquatic foods; Fig. 4) will increase our understanding on the current and future role of aquatic foods for nutrition [53].

### Improving Aquatic Food Nutrient Composition Inference

Better baseline data will allow to further improve nutrient composition predictability and inference. For example, models that build upon observed nutrient concentration data to predict the nutrient concentration of fish raw muscle tissue based on life-history traits (e.g., [7•]) or phylogeny (e.g., [6]) are useful tools to infer nutrient composition for species lacking such information. However, such inferences need to be expanded to include (i) other nutrients important for public health such as vitamin B_12_ or vitamin D (Fig. 2), (ii) other aquatic food groups besides ray-finned fishes that are important nutritionally (e.g., invertebrates or aquatic plants; e.g., Fig. 4), (iii) spatio-temporal variability within species groups [53, 58], and (iv) nutrition variability among consumed body parts, production sectors and/or preparation methods. Such advancements will increase the accuracy of nutrient predictions and inform policy agendas aimed at minimizing the burden of malnutrition through aquatic food management (e.g., [59]).

### Improving the Accuracy of Target Population Needs

There is a lack of complete, accurate, and high-quality data on micronutrient intake and malnutrition around the globe [34], limiting our understanding on population needs and requirements, and in turn which nutrients we need to prioritize. For example, many essential nutrients (e.g., Fig. 2) are lacking inadequate intake information (Fig. 3), dietary references such as the estimated average requirements (EAR) [24], and/or accurate deficiency estimates based only on access to foods (e.g., zinc intake from national food supplies compared to biological outcomes of zinc deficiency [34, 60]). Assessing the nutritional needs of the target population will require better compilation of multiple evidence sources (e.g., biological, clinical, or functional markers; nutrient adequacy of individual diets; nutrient adequacy of household diets; nutrient adequacy of national food supplies; and nutrient-informative food-group intake of individuals or households; [35]), and different bioavailability and absorption rates. Compiling accurate population deficiencies will better inform aquatic food research needs.

### Understanding Bioavailability and Nutrient Interactions

Distinctions between nutrient bioavailability and absorption with particular relevance for aquatic food research and which nutrients to select also exist. For example, total iron is typically broken up into heme iron (animal-sourced) and non-heme iron (plant-sourced) with varying absorption rates in the human body [61]. Other compounds such as phytates inhibit the absorption of iron, calcium, and zinc [62]. Additionally, metabolic interactions between vitamins and minerals determine their physiologic utility and intake requirements. For example, the mineral calcium is necessary for healthy cardiovascular and skeletal systems. Yet, absorption of calcium from the diet is strongly dependent on the vitamin D derivative calcitriol. In conditions of vitamin D deficiency, the body draws upon calcium stores from the skeletal system, increasing the risk of osteoporosis and other degenerative bone diseases [23••]. Understanding such nutrient interdependencies and considering them in nutrient selection (e.g., which nutrients to combine together to maximize absorption) may be crucial when focusing on aquatic foods, and their combined benefit to nutrition.

### Accounting for Aquatic Foods as a Whole

Foods provide a combination of nutrients simultaneously. Sometimes, narrowing the picture to individual nutrients instead of the overall pool of nutrients that foods may provide (as is the case in current nutrient supply analyses) can misrepresent the combined nutritional value of aquatic foods and/or other foods and thus the contribution of aquatic foods to healthy diets relative to other food sources. Several combined metrics are being explored that include several nutrients or several nutrients relative to nutrient reference intakes (e.g., [11•, 53]). However, as nutrient selection can influence study outcomes, “which” nutrients are combined, “how” and “why” requires further research attention. Testing metrics that combine nutrients based on physiological relevance (e.g., to prevent non-communicable diseases), consider nutrients that must be consumed simultaneously (e.g., to improve absorption; [63]), and/or also take into consideration the nutrition qualities of other foods that are ingested together with aquatic foods (e.g., account for dietary patterns) will help provide a better picture of the importance of aquatic foods for nutrition security as a whole.

### Expanding the Scope of Aquatic Food Attributes to Include Public Health Risks and Environmental Footprint

Considering aquatic foods as a beneficial nutrient pool, while important, could mask potential risks (e.g., contaminants and toxicants, allergies or microbial pathogens) associated with aquatic food consumption such as the accumulation of heavy metals (e.g., mercury), microplastics, or polychlorinated biphenyls (PCBs) that are toxic for the human body [64, 65]. Likewise, food production has a big environmental impact, with aquatic systems estimated to contribute 9.9% of the pressures of the global food system [66]. A better understanding of the trade-offs between nutritional benefits, upper intake levels, pathogens, contaminant risks, and environmental footprints relative to other food sources will allow researchers and practitioners to add extra dimensions to nutrient selection, set better spatially and species-specific safe consumption limits, and inform holistic management approaches that maximize nutritional benefits.

## Conclusion

Aquatic foods are a rich and diverse source of macro and micronutrients, highlighting the potential role that fisheries and aquaculture sectors may serve in preventing malnutrition. To understand the role of aquatic foods for nutrition security, researchers must first identify the set of nutrients to target for analysis or optimization. Our review proposes a framework to select nutrients based on physiological importance, needs of the study population, and relevance of aquatic foods relative to other foods accessible in the food system. We show that there are at least 41 essential nutrients obtained from the diet and that the best pathways to target malnutrition with aquatic foods will depend on how each of these nutrients is deficient in the study population, as well as their total availability in the food system. Obtaining spatio-temporal nutrient composition and deficiency data on these 41 nutrients, as well as increasing our understating of their bioavailability and interactions, will further contribute towards an understanding of the nutritive role of aquatic foods, informing fisheries management and aquaculture initiatives aimed at decreasing the burden of malnutrition and improving public health.

## Methodology

### Identifying Nutrients Important for Public Health

We conducted targeted searches on the Web of Science using keywords nutrient*, *public health*, *essential*, *micronutrient*, *vitamin*, and *minerals* to identify studies for a literature review. Our initial screening on the Web of Science using keywords nutrient*, *public health*, and *essential* returned 469 studies, from which we selected 26 sources of peer-reviewed literature and scientific reports that summarized or provided a review of data from clinical trials and lab-based studies. Additional searches were conducted using keywords *micronutrient*, *vitamin*, and *minerals* and specific nutrient names. We summarized the literature to create a comprehensive list of nutrients important for public health through dietary consumption. From these nutrients, we created a list of essential nutrients based on the most recent scientific evidence of nutrient deficiencies, nutrients with severe consequences when under-consumed, and nutrients of public health importance (Table S1).

### Global Inadequate Intake

To determine global inadequate intakes as an example of nutritional needs of populations, we used four global studies that had estimated inadequate intake: Beal et al. 2017; Passarelli et al. 2022; Zhou and Liang 2021; Golden et al 2021 [i.e., 33, 37, 38, 4•]. We averaged the deficiency value from different sources to determine the final deficiency by nutrient and country.

### Nutrient Concentration from Aquatic Foods and Other Food Groups

To exemplify the importance of accounting for aquatic foods relative to other foods accessible in the food system in terms of nutrient concentration, we used the Aquatic Food Composition Database [47] for nutrient composition of aquatic foods, and USDA National Nutrient Database (USDA) [48] for nutrient composition of all other foods. The Aquatic Food Composition Database synthesizes nutrient information from 26 national and international food composition tables and over 950 peer review studies into a single database containing over 2500 taxa and 300 nutrients along with data on samples, including sample origin, sample preparation, and part of aquatic food analyzed. All units were standardized to FAO INFOODs guidelines. Aquaculture feeding trials were excluded from the dataset. A quality check was conducted to identify outliers and make sure units and values were correct. All taxonomic information was standardized according to FishBase [18] and SeaLifeBase [67] taxonomic tables. The US Department of Agriculture (USDA) National Nutrient Database for Standard Reference is the major source of food composition data in the United States and provides the foundation for most food composition databases in the public and private sectors in the US.

To compare nutrient composition of different food groups, we first standardized all units for accessed nutrients across AFCD and USDA databases. For USDA, we used the food categories from the database to calculate the median nutrient values of each food group. We used only raw products for all databases (excluding cooked products) and for AFCD we used only the muscle tissue of aquatic species (excluding viscera, bones, head, tail, etc.). To compare across different food groups, we used the median value of each food group relative to the maximum median nutrient value across all groups. To compare nutrient content across different aquatic food species groups (ray-finned fish, sharks and rays, molluscs, crustaceans, and seaweed) we used the median value of each taxonomic group relative to the median value across all aquatic food groups.

Note that we also performed the analyses using only the USDA data instead of AFCD, which has less diversification of aquatic food groups, yet trends in concentrations relative to other food groups were consistent for all nutrients except for those without information (Fig. S2).

### Nutrient Apparent Consumption

To provide an empirical example of the importance of accounting for quantity of aquatic foods relative to other food sources, we estimated the contribution of aquatic foods to nutrients in terms of quantity using an updated version (year 2017) of apparent consumption estimates from the Global Nutrient Database (GND)[49••]. GND contains information on per capita daily apparent consumption of 156 nutrients across 195 countries and territories separated by food and agricultural commodity groups. We filtered the database for the nutrients highlighted in Fig. 2, and for each nutrient with available information, we calculated the median per capita daily apparent consumption (across all countries) obtained from all foods and obtained only from aquatic foods. Next, for each nutrient, we calculated the proportion of per capita daily apparent consumption obtained from aquatic foods by dividing the quantity obtained from aquatic foods by the quantity obtained from all foods.

## Supplementary Information

Below is the link to the electronic supplementary material.

Supplementary file1 (DOCX 1.07 MB)

## Data Availability

Data used for this paper are available from the following references: [4•, 33, 37, 38, 47, 48]. Apparent consumption estimates [49] related to this paper may be requested from the author [JS] upon reasonable request.

## Declarations

### Disclaimer

The views expressed in this article by Dr. Passarelli are solely the personal views of this author.
