# Supplementary material for: A Decision Framework for Selecting Critically Important Nutrients from Aquatic Foods
Source: Curr Environ Health Rep. 2023 May 25;10(2):172–83. doi: 10.1007/s40572-023-00397-5 (PMC10299940; doi:10.1007/s40572-023-00397-5)
Supplement: Supplementary file 1 — Supplementary file1 (DOCX 1.07 MB) [file 40572_2023_397_MOESM1_ESM.docx]

**Supplemental information**

**
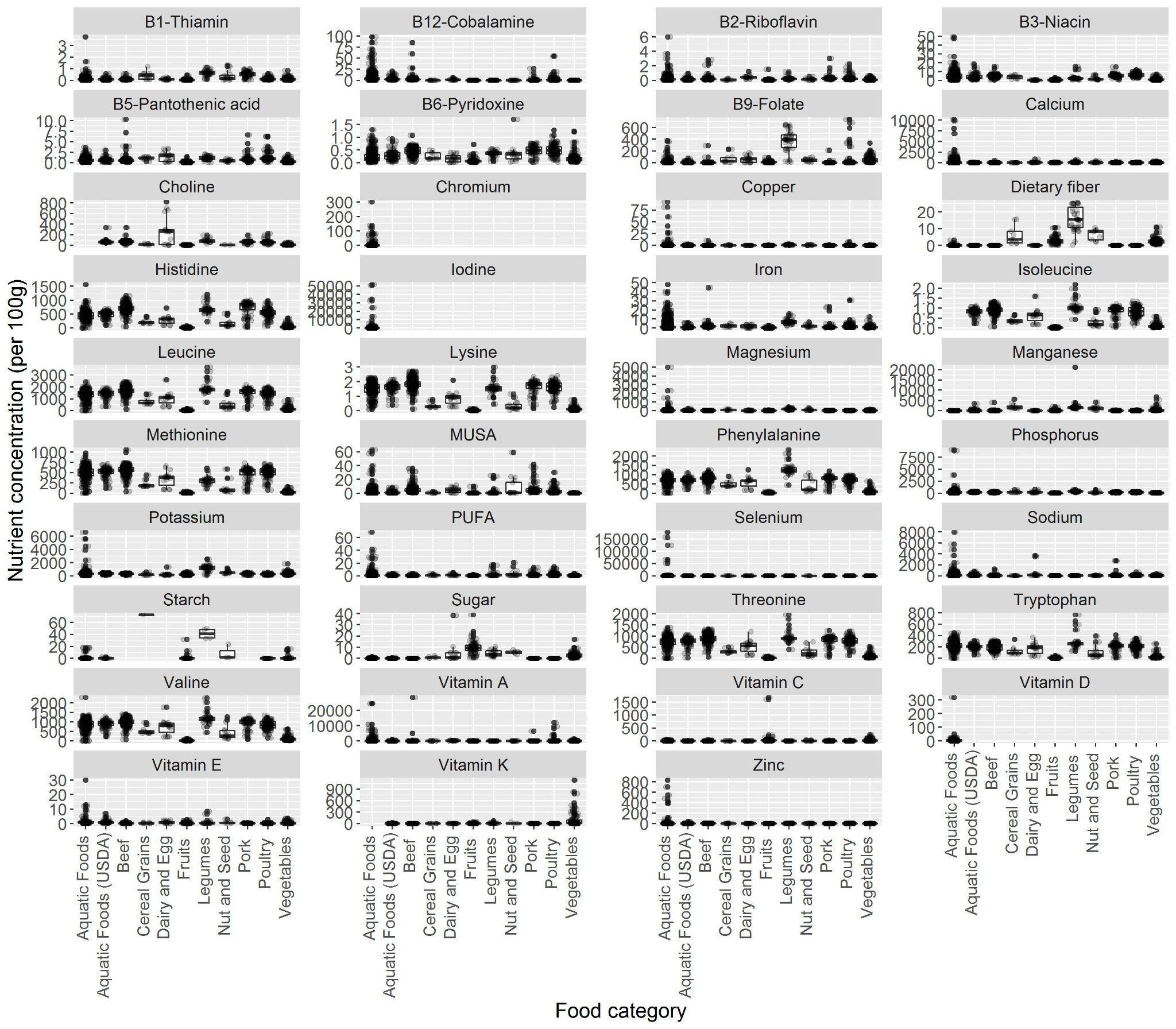
**

**Figure S1| Within food group variability in nutrient concentrations.** Each point is a food with available nutrient information.

**
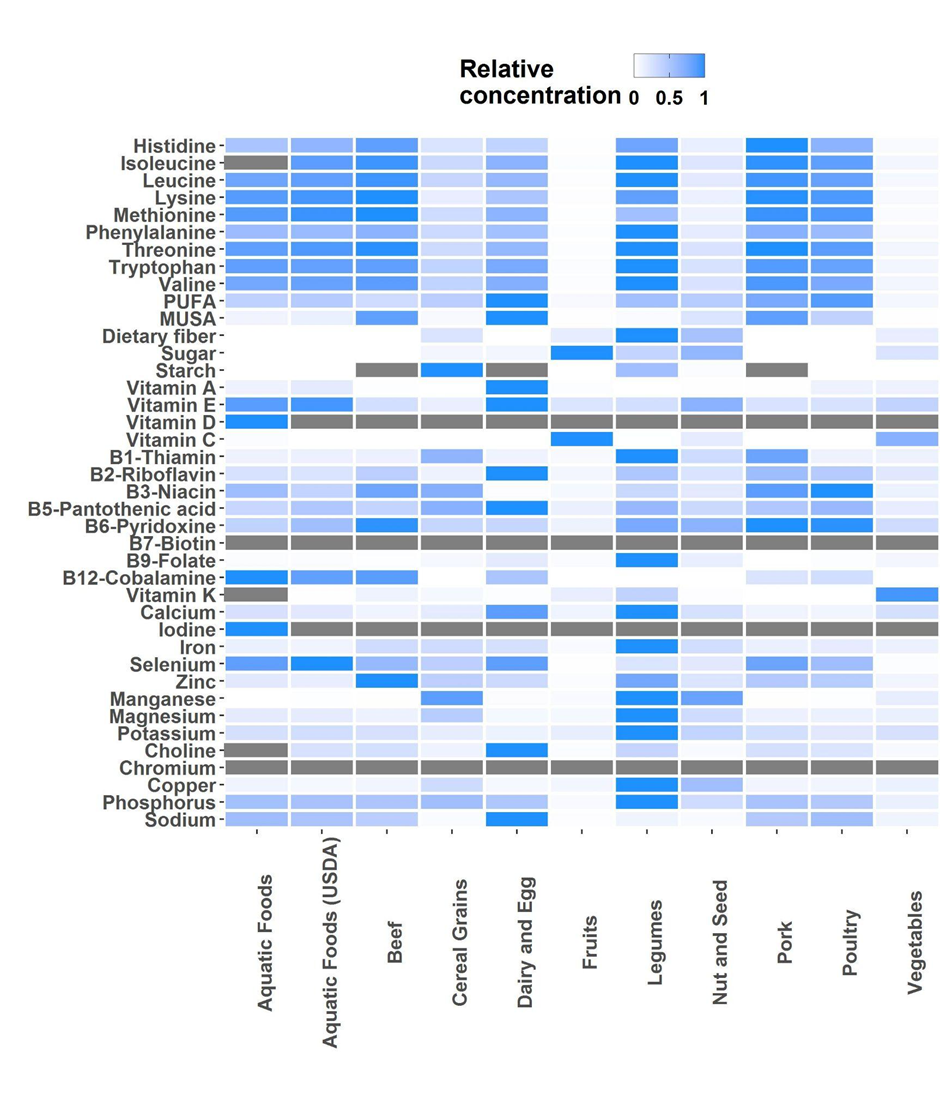
Figure S2| Sensitivity check on using AFCD or only USDA to get aquatic food concentration estimates.** Note that except for those nutrients that are missing in one of the databases, aquatic foods rank similarly among databases.

**Table S1| Identified key nutrients for physiological functioning from dietary sources.** Nutrients are divided into macronutrients and micronutrients. We state the body function they contribute to, their deficiency impact, their top food sources (with a maximum of six if more food sources were stated) and the relevant literature. Note that blue shading in some nutrients indicates that some type of aquatic food was highlighted as one of the main food sources.

| **Nutrient** | Body function | | | Deficiency impact | Food sources | Ref. |
| --- | --- | --- | --- | --- | --- | --- |
| **Macronutrients** |  | | |  |  |  |
| **Protein (total)** | Structural components of all cells; function as enzymes, membrane carriers and hormones; repair body tissue; break down food; growth and development | | | Sarcopenia; reduced muscle strength; impaired growth and development; impaired immune function; thin hair; dull skin; edema | Meat; seafood; eggs; dairy; legumes; nuts and seeds | (Dietary Guidelines Advisory Committee, 2020 [68]; Solan, 2021 [69]) |
| Essential amino acids: Histidine, Isoleucine, Leucine, Lysine, Methionine, Phenylalanine, Threonine, Tryptophan, Valine | Development and growth; protein synthesis | | | Shift in the balance of protein synthesis and protein degradation | Meat; seafood; dairy | (Berrazaga et al., 2019 [70]; Wu, 2009 [71]) |
| **Carbohydrates** | Provide energy to cells | | | Fatigue; weakness; constipation; reduced central nervous system function and development; micronutrient deficiency | Grains; fruits; vegetables; processed foods | (Dietary Guidelines Advisory Committee, 2020 [68]; Panel on Macronutrients, 2005 [72]) |
| Dietary fiber (total)* | Delay gastric emptying of foods into small intestine for feeling of fullness; slow absorption of fat and cholesterol | | | Increased risk of cardiovascular disease and colon cancer | Fruits; vegetables; legumes; whole grains; nuts |  |
| Sugar | Provides energy to cells | | | See Carbohydrates deficiency impact | Fruit; processed drinks; food |  |
| Starch | Provides longer term energy to cells | | | See Carbohydrates deficiency impact | Grains; legumes; tubers; corn |  |
| **Fat** | Major source of fuel energy for the body; aids in absorption of fat-soluble vitamins; provides structural and metabolic functions | | | Impaired growth; increased risk of disease; fatigue; muscle wasting | Butter & oils; fatty meat; whole milk; egg yolk; nuts | (Dietary Guidelines Advisory Committee, 2020 [68]) |
| Monounsaturated fatty acids | Improve blood cholesterol levels; reduce inflammation; decrease risk of coronary artery disease | | | Increased risk of heart disease | Canola oil; olive oil; high-oleic sunflower oil; high-oleic safflower oil; meat fat | (White, 2009 [73]) |
| Polyunsaturated fatty acids: omega 6 | Inflammatory mediation; gene expression; growth; oxygen transport; energy storage; potential protection against dementia | | | Rough and scaly skin; hair loss; impaired wound healing; increased susceptibility to infection; reduced growth in children | Nuts; seeds; vegetable oils (sunflower, safflower, corn soybean oils) | (Genuis & Schwalfenberg, 2006 [74]; Gramlich et al., 2019 [75]; Panel on Macronutrients et al., 2005 [72]) |
| Polyunsaturated fatty acids: omega 3 | Development of placenta; development of fetus; development of brain | | |  | Flaxseed; canola and soybean oils; fatty fish; fish oil |  |
| **Micronutrients** | | | | | | |
| **Calcium*** | Builds bone and teeth; cell signaling; muscle and nerve function; regulated vasodilation and constriction | | | Low bone mass; osteoporosis; gestational hypertension; preterm birth | Dairy; dark leafy greens; seafood; tofu; seeds | (Beal & Ortenzi, 2022 [36]; Panel on Macronutrients et al., 2005 [72]) |
| **Iron**~^** | Transports oxygen to cells; immune response by differentiation and growth of epithelial tissue | | | Anemia; cognitive impairment | Red meats & organs; poultry; seafood; dark leafy greens; fruit | (Beal & Ortenzi, 2022 [36]; Dietary Guidelines Advisory Committee, 2020 [68]; Gombart et al., 2020 [76]) |
| **Magnesium~** | Muscle contraction, muscle building; nerve transmission; immune support | | | Neurological or neuromuscular impacts including muscle cramps, seizures, vertigo, muscle weakness; fatigue; lethargy; staggering; loss of appetite; decreased resistance to infection | Green leafy vegetables; legumes; whole grains; nuts and seeds | (Calder et al., 2020 [77]; Dietary Guidelines Advisory Committee, 2020 [68]; Gombart et al., 2020 [76]) |
| **Manganese** | Bone formation; reactions related to amino acids; cholesterol and carbohydrate metabolism | | | Scaly dermatitis; low cholesterol; impaired growth and skeletal development | Grains; tea; vegetables | (National Academies, 2006 [78]) |
| **Phosphorus** | Bone mineralization; healthy aging; transfer of energy | | | Bone loss; cardiac and respiratory failure; anemia; anorexia | Dairy; soft drinks | (Dietary Guidelines Advisory Committee, 2020 [68]; National Academies, 2006 [78]; Serna & Bergwitz, 2020 [79]) |
| **Potassium*** | Cellular function; regulation of intracellular fluid and electrolyte balance; transmembrane electrochemical gradients | | | Hypokalemia, which causes cardiac arrhythmias; muscle weakness and glucose intolerance; hypertension; salt sensitivity; kidney stones | Fruits and vegetables; seafood; dairy | (National Academies of Sciences, 2019 [80]) |
| **Sodium** | Maintenance of extracellular fluid volume and plasma osmolality | | | Hyponatremia; altered personality; lethargy; confusion; seizures; death | Salt (sodium chloride); processed foods | (Bailey et al., 2021 [81]; Dietary Guidelines Advisory Committee, 2020 [68]; National Academies of Sciences, 2019 [80]) |
| **Zinc^** | Essential cofactor for enzymatic reactions; immune support; maintains integrity of skin and mucosal membrane; protein synthesis; cell division | | | Poor health; increased risk of diarrhea; impaired cognitive and motor development; preterm births; reduced immunity; impaired wound healing; acrodermatitis enterohepatica | Muscle and organ meat; seafood; eggs; dairy; seeds | (Beal et al., 2021 [35]; Beal & Ortenzi, 2022 [36]; Gombart et al., 2020 [76]) |
| **Choline** | Neurotransmitter synthesis; cell-membrane signaling; lipid transport; brain and memory development in fetus | | | Organ dysfunction in adults; liver damage; neural tube defects and cognitive impairments in fetus; increased risk of cardiovascular disease | Meat; seafood; poultry; dairy; eggs | (Beal & Ortenzi, 2022 [36]; National Academies, 2006 [78]; Zeisel & da Costa, 2009 [82]) |
| **Chromium** | Improves the action of insulin | | | Glucose intolerance | Whole grains; meat; poultry; seafood | (Capone & Sentongo, 2019 [83]; Maret, 2019 [84]) |
| **Copper** | Immune support; energy metabolism; iron metabolism; antioxidation | | | Anemia; osteoporosis; low white blood cell count; thyroid problems | Organ meats; seafood; nuts and seeds; whole grains; cocoa | (Gombart et al., 2020 [76]; Capone & Sentongo, 2019 [83]) |
| **Selenium** | Immune support; thyroid function; antioxidation | | | Hair and skin depigmentation; macrocytosis; muscle weakness; cardiomyopathy; genetic mutations and increased virulence of viruses | Meat; seafood; cereals and grains; dairy; fruits and vegetables | (Calder et al., 2020 [77]; Gombart et al., 2020 [76]; Hu et al., 2021 [19]; Capone & Sentongo, 2019 [83]; US Department of Health and Human Services, nd [85]; National Academies, 2006 [78]) |
| **Iodine**^** | Neurological development and fetal growth | | | Impaired neurological and irreversible behavioral development in child; goiter; death; cretinism in fetus | Seafood; processed foods with additives; iodized salt | (Dietary Guidelines Advisory Committee, 2020 [68]) |
| **Vitamin A~^** | Normal differentiation of epithelial tissue; intestinal immune response to support the gut barrier; immunoregulatory functions | | | Night blindness; increased susceptibility to infections; death | Seafood; dark leafy greens; red/orange vegetables; animal dairy; organ meat; eggs | (Beal et al., 2021 [35]; Beal & Ortenzi, 2022 [36]; Black et al., 2013 [86]; Gombart et al., 2020 [76]) |
| **Vitamin E~** | Protects cell membranes from damage caused by free radicals; supports epithelial barriers | | | Genetic mutations; increased virulence of viruses | Vegetable oils; avocados; nuts | (Calder et al., 2020 [77]; Gombart et al., 2020 [76]) |
| **Vitamin D*** | Aids in absorption of calcium for bone health; immune support; fetal development | | | Osteoporosis; autoimmune diseases; cancer; hypertension; severe pre-eclampsia in pregnant women; preterm birth; low birthweight infant | Fatty fish; eggs from hens fed vitamin D and/or exposed to sunlight | (Bendik et al., 2014 [87]; Black et al., 2013 [86]; Gombart et al., 2020 [76]) |
| **Vitamin C~** | Cofactor for metalloenzymes; peptide hormones synthesize; gene regulation; immune response; growth and development of infants and children | | | Fatigue; lethargy; mood changes; scurvy; increased risk of cancer and CVD; respiratory disease | Citrus fruits; tomatoes; potatoes; leafy green vegetables; brassicas | (Gombart et al., 2020 [76]; Rowe & Carr, 2020 [88]) |
| **Thiamine (Vitamin B1)** | Coenzyme in the metabolism of carbohydrates and amino acids | | | Beriberi disease; wet edema; dry muscle wasting;  Wenicke-Korsakoff syndrome; cardiovascular complications;  weight loss; mental changes; heart failure; neuropathy; encephalopathy | Fortified grains; pork; eggs; legumes; nuts | (Bruins et al., 2019 [89]; Lonsdale, 2006 [90]; National Academies, 2006 [78]) |
| **Riboflavin (Vitamin B2)** | Coenzyme for essential cellular processes including mitochondrial energy metabolism, stress response, vitamin and cofactor biogenesis | | | Sore throat; skin inflammation; swollen tongue; anemia; impaired nerve function | Eggs; organ meats; lean meats; dairy; seafood; fortified grains | (LeBlanc et al., 2011 [91]; Mosegaard et al., 2020 [92]; National Academies, 2006 [78]) |
| **Niacin (vitamin B3)** | neuronal development and survival | | | Diarrhea, dermatitis, and dementia (the three D Disease); Increased risk of neurodegenerative diseases | Yeast; meat; seafood; legumes; fortified grains | (Gasperi et al., 2019 [93]) |
| **Vitamin B5 (pantothenic acid)** | Synthesis of coenzyme A; involved in fatty acid metabolism; synthesis of vitamin A & D | | | Irritability and restlessness; fatigue; apathy; sleep disturbance; neurobiological symptoms | Chicken; beef; organ meat; whole grains; mushrooms; avocados | (National Academies, 2006 [80]; US Department of Health and Human Services, nd [94]) |
| **Vitamin B6 (pyridoxine)** | Cognitive function; intestinal immune regulation to support the gut barrier; coenzyme for metabolism of amino acids | | | Cognitive decline; depression; neurologic disorders | Fruits; vegetables; meat; seafood; dairy; fortified grains | (Dietary Guidelines Advisory Committee, 2020 [68]; Gombart et al., 2020 [76]) |
| **Vitamin B7 (Biotin)** | Coenzyme for carboxylation reactions | | | Dermatitis around the mouth, nose and eyes; alopecia; hypotonia; ataxia | Liver; eggs; meat; nuts and seeds; avocados | (Capone & Sentongo, 2019 [83]) |
| **Folate (vitamin B9)**~^+** | Neural tube development in fetus; intestinal immune regulation to support the gut barrier; cognitive functioning | | | In pregnant women neural tube defects in fetus; in infants and young children anemia, hindered brain development, adult depression | Dark leafy greens; legumes; organ meat; fruit; seafood; eggs | (Beal et al., 2021 [35]; Beal & Ortenzi, 2022 [36]; Gombart et al., 2020 [76]) |
| **Vitamin B12 (cobalamine)^** | Cognitive function; intestinal immune regulation to support the gut barrier | | | Anemia; development regression; depression; cognitive impairment | Organ meats; seafood; red meat; dairy; eggs; poultry | (Beal & Ortenzi, 2022 [36]; Dietary Guidelines Advisory Committee, 2020 [68]; Gombart et al., 2020 [76]) |
| **Vitamin K** | Musculoskeletal health through bone matrix mineralization; coagulation | | | Low bone mass; osteoporosis; bleeding; easy bruising | Green leafy vegetables; vegetable oils | (Bruins et al., 2019 [89]; Capone & Sentongo, 2019 [83]) |
|  |  |  |  |  |  |  |

**Supplementary references**

68. Dietary Guidelines Advisory Committee. *Scientific Report of the 2020 Dietary Guidelines Advisory Committee: Advisory Report to the Secretary of Agriculture and Secretary of Health and Human Services*. U.S. Department of Agriculture, Agricultural Research Service; 2020. https://doi.org/10.52570/DGAC2020

69. Solan M. The best foods for vitamins and minerals. Harvard Health Publishing. Published August 17, 2021. https://www.health.harvard.edu/staying-healthy/the-best-foods-for-vitamins-and-minerals

70. Berrazaga I, Micard V, Gueugneau M, Walrand S. The Role of the Anabolic Properties of Plant- versus Animal-Based Protein Sources in Supporting Muscle Mass Maintenance: A Critical Review. *Nutrients*. 2019;11(8):1825. https://doi.org/10.3390/nu11081825

71. Wu G. Amino acids: metabolism, functions, and nutrition. *Amino Acids*. 2009;37(1):1-17. https://doi.org/10.1007/s00726-009-0269-0

72. Panel on Macronutrients, Panel on the Definition of Dietary Fiber, Subcommittee on Upper Reference Levels of Nutrients, et al. *Dietary Reference Intakes for Energy, Carbohydrate, Fiber, Fat, Fatty Acids, Cholesterol, Protein, and Amino Acids*. National Academies Press; 2005:10490. https://doi.org/10.17226/10490

73. White B. Dietary fatty acids. *Am Fam Physician*. 2009;80(4):345-350.

74. Genuis SJ, Schwalfenberg GK. Time for an oil check: the role of essential omega-3 fatty acids in maternal and pediatric health. *J Perinatol*. 2006;26(6):359-365. https://doi.org/10.1038/sj.jp.7211519

75. Gramlich L, Ireton‐Jones C, Miles JM, Morrison M, Pontes‐Arruda A. Essential Fatty Acid Requirements and Intravenous Lipid Emulsions. *J Parenter Enter Nutr*. 2019;43(6):697-707. https://doi.org/10.1002/jpen.1537

76. Gombart AF, Pierre A, Maggini S. A Review of Micronutrients and the Immune System–Working in Harmony to Reduce the Risk of Infection. *Nutrients*. 2020;12(1):236. https://doi.org/10.3390/nu12010236

77. Calder P, Carr A, Gombart A, Eggersdorfer M. Optimal Nutritional Status for a Well-Functioning Immune System Is an Important Factor to Protect against Viral Infections. *Nutrients*. 2020;12(4):1181. https://doi.org/10.3390/nu12041181

78. National Academies. *Dietary Reference Intakes: The Essential Guide to Nutrient Requirements*. National Academies Press; 2006:11537. https://doi.org/10.17226/11537

79. Serna J, Bergwitz C. Importance of Dietary Phosphorus for Bone Metabolism and Healthy Aging. *Nutrients*. 2020;12(10):3001. https://doi.org/10.3390/nu12103001

80. National Academies of Sciences, Food and Nutrition Board, Health and Medicine Division, National Academies of Sciences, Engineering, and Medicine. *Dietary Reference Intakes for Sodium and Potassium*. (Stallings VA, Harrison M, Oria M, eds.). National Academies Press; 2019:25353. https://doi.org/10.17226/25353

81. Bailey RL, Ard JD, Davis TA, et al. A Proposed Framework for Identifying Nutrients and Food Components of Public Health Relevance in the Dietary Guidelines for Americans. *J Nutr*. 2021;151(5):1197-1204. https://doi.org/10.1093/jn/nxaa459

82. Zeisel SH, da Costa KA. Choline: an essential nutrient for public health. *Nutr Rev*. 2009;67(11):615-623. https://doi.org/10.1111/j.1753-4887.2009.00246.x

83. Capone K, Sentongo T. The ABCs of Nutrient Deficiencies and Toxicities. *Pediatr Ann*. 2019;48(11). https://doi.org/10.3928/19382359-20191015-01

84. Maret W, ed. Chromium Supplementation in Human Health, Metabolic Syndrome, and Diabetes. *Essent Met Med Ther Use Toxic Met Ions Clin*. Published online January 14, 2019. https://doi.org/10.1515/9783110527872-015

85. US Department of Health and Human Services. Selenium—health professional fact sheet. Accessed December 18, 2022. https://ods.od.nih.gov/factsheets/Selenium-HealthProfessional/

86. Black RE, Victora CG, Walker SP, et al. Maternal and child undernutrition and overweight in low-income and middle-income countries. *The Lancet*. 2013;382(9890):427-451. https://doi.org/10.1016/S0140-6736(13)60937-X

87. Bendik I, Friedel A, Roos FF, Weber P, Eggersdorfer M. Vitamin D: a critical and essential micronutrient for human health. *Front Physiol*. 2014;5. https://doi.org/10.3389/fphys.2014.00248

88. Rowe S, Carr AC. Global Vitamin C Status and Prevalence of Deficiency: A Cause for Concern? *Nutrients*. 2020;12(7):2008. https://doi.org/10.3390/nu12072008

89. Bruins MJ, Van Dael P, Eggersdorfer M. The Role of Nutrients in Reducing the Risk for Noncommunicable Diseases during Aging. *Nutrients*. 2019;11(1):85. https://doi.org/10.3390/nu11010085

90. Lonsdale D. A Review of the Biochemistry, Metabolism and Clinical Benefits of Thiamin(e) and Its Derivatives. *Evid Based Complement Alternat Med*. 2006;3(1):49-59. https://doi.org/10.1093/ecam/nek009

91. LeBlanc JG, Laiño JE, del Valle MJ, et al. B-Group vitamin production by lactic acid bacteria - current knowledge and potential applications: Vitamin production by LAB. *J Appl Microbiol*. 2011;111(6):1297-1309. https://doi.org/10.1111/j.1365-2672.2011.05157.x

92. Mosegaard S, Dipace G, Bross P, Carlsen J, Gregersen N, Olsen RKJ. Riboflavin Deficiency—Implications for General Human Health and Inborn Errors of Metabolism. *Int J Mol Sci*. 2020;21(11):3847. https://doi.org/10.3390/ijms21113847

93. Gasperi V, Sibilano M, Savini I, Catani M. Niacin in the Central Nervous System: An Update of Biological Aspects and Clinical Applications. *Int J Mol Sci*. 2019;20(4):974. https://doi.org/10.3390/ijms20040974

94. US Department of Health and Human Services. Pantothenic Acid—Health Professional Fact Sheet. Accessed December 18, 2022. https://ods.od.nih.gov/factsheets/PantothenicAcid-HealthProfessional/
